# Disease alleviating effects following prophylactic lemon and coriander essential oil treatment in mice with acute campylobacteriosis

**DOI:** 10.3389/fmicb.2023.1154407

**Published:** 2023-03-29

**Authors:** Soraya Mousavi, Dennis Weschka, Stefan Bereswill, Markus M. Heimesaat

**Affiliations:** Gastrointestinal Microbiology Research Group, Institute of Microbiology, Infectious Diseases and Immunology, Charité - Universitätsmedizin Berlin, Corporate Member of Freie Universität Berlin, Humboldt-Universität zu Berlin, and Berlin Institute of Health, Berlin, Germany

**Keywords:** lemon essential oil (citrus Limon l.), coriander essential oil, *Campylobacter jejuni*, secondary abiotic IL-10−/− mice, campylobacteriosis model, host-pathogen interaction, immune-modulatory effects, natural antibiotics-independent compounds

## Abstract

**Introduction:**

Given the worldwide increasing prevalence of human *Campylobacter jejuni* infections and the emergence of multi-drug resistant enteropathogenic strains, antibiotic-independent approaches applying non-toxic natural compounds for the treatment and prophylaxis of campylobacteriosis appear utmost desirable. In our placebo-controlled intervention study, we surveyed potential disease-alleviating including anti-pathogenic and immune-modulatory effects upon prophylactic oral application of lemon-essential oil (LEM-EO) and coriander-essential oil (COR-EO) in acute experimental campylobacteriosis.

**Methods:**

Therefore, secondary abiotic IL-10^−/−^ mice were orally challenged with either LEM-EO or COR-EO starting seven days prior to peroral *C. jejuni* infection.

**Results and discussion:**

Six days post-infection, slightly lower pathogen loads were assessed in the colon of mice from the LEM-EO as opposed to the COR-EO cohort if compared to placebo counterparts. Prophylactic application of both EOs improved the clinical outcome of acute campylobacteriosis which was paralleled by less distinct pathogen-induced colonic epithelial cell apoptosis. Moreover, mice subjected to LEM-EO and COR-EO prophylaxis displayed lower colonic numbers of macrophages/monocytes and of T lymphocytes, respectively, whereas in both verum groups, basal IL-6 and IFN-γ concentrations were measured in mesenteric lymph nodes on day 6 post-infection. The oral challenge with either EOs resulted in diminished secretion of distinct pro-inflammatory mediators in the kidney as well as serum samples derived from the infected mice. In conclusion, the results from our preclinical *in vivo* study provide evidence that LEM-EO and COR-EO constitute promising prophylactic measures to prevent severe campylobacteriosis which may help to reduce the risk for development of post-infectious sequelae in *C. jejuni* infected individuals.

## 1. Introduction

*Campylobacter jejuni* is a Gram-negative food-borne pathogen leading to millions of enteric infections worldwide (EFSA, 2021). Whereas *C. jejuni* cause acute gastroenteritis in infected humans, these bacteria are commensals in various species of livestock such as poultry (WHO, 2020). After the ingestion of undercooked, contaminated animal products or surface water, the bacteria cross the mucosal surface of intestinal crypts and induce immune responses that are caused by the Gram-negative bacterial cell wall molecule lipo-oligosaccharide (LOS) subsequently resulting in inflammation (Callahan et al., 2021). As a Toll-like receptor-4 (TLR-4) agonist, LOS induces hyperactivation of the innate and adaptive immune system *via* TLR-4 and the mammalian target of rapamycin (mTOR) signaling (Sun et al., 2012). Infected patients commonly complain about symptoms of acute gastroenteritis, such as fever, abdominal pain, and bloody diarrhea (Walker et al., 1986; Facciolà et al., 2017). Most acute *C. jejuni* infections are self-limiting and require symptomatic interventions, such as pain relief and electrolyte substitution. On rare occasions, however, post-infectious autoimmune diseases, such as Guillain-Barré syndrome, reactive arthritis, and intestinal morbidities, including inflammatory bowel diseases, irritable bowel syndrome and celiac disease are observed with a latency of weeks to months after the primary infectious event (Keithlin et al., 2014; Zautner et al., 2014). Importantly, the severity of initial enteritis has been shown to be associated with the risk for the development of post-infectious sequelae (Mortensen et al., 2009). Therefore, severe and invasive *C. jejuni* induced gastroenteritis in otherwise compromised patients may require antibiotic therapy. However, the progressive development of drug resistance by the enteropathogens against the most commonly used antibiotic options such as quinolones and macrolides limit the successful anti-pathogenic treatment of human campylobacteriosis if required (Mouftah et al., 2021). Therefore, natural plant-derived compounds including essential oils (EOs) with anti-microbial and immune-modulatory properties may constitute alternative strategies to treat severely compromised patients with acute campylobacteriosis and limit the risk of antibiotic resistance development. Particularly lemon (LEM) and coriander (COR) EOs have gained increasing scientific interest in the combat of food-borne pathogens recently (Rattanachaikunsopon and Phumkhachorn, 2010; Micciche et al., 2018, 2019; Salem et al., 2019).

*Citrus limon* belongs to the Rutaceae family and has previously been reported to possess anti-microbial and anti-inflammatory including anti-oxidant properties (Rozza et al., 2011). The Food and Drug Administration (FDA) has rated LEM as generally safe and therefore, it constitutes one of the main sources of EO used as preservative in the food industries. *In vitro* studies revealed that LEM-EO, with limonene as the main constituent, exerts potent anti-microbial effects directed against Gram-positive bacteria such as *Staphylococcus aureus*, *Enterococcus faecalis*, *Bacillus subtilis*, and against Gram-negative bacterial species including *Klebsiella pneumoniae* and *Salmonella* Paratyphi A (Schieberle and Grosch, 1988; Mehmood et al., 2019; Yazgan et al., 2019; Pucci et al., 2020). Furthermore, pretreatment with LEM-EO was shown to down-regulate the expression of lipo-polysaccharide (LPS) induced pro-inflammatory cytokines such as tumor necrosis factor-alpha (TNF-α) and interleukin (IL)-1β and IL-6 (Yoon et al., 2010; Pucci et al., 2020) in murine and human macrophages underlining the potent anti-inflammatory effects of LEM-EO.

*Coriandrum sativum* L. belongs to the Apiaceae family and is widely used as flavoring agent in food and cosmetics (Varier, 1994). Due to its anti-inflammatory properties, COR seeds have been used as a treatment option of inflammatory diseases such as rheumatism (Varier, 1994). Furthermore, COR attenuated liver ischemia-induced TNF-α production and apoptotic cell death of hepatocytes in rats (Kükner et al., 2021). COR-EO with linalool and linalyl acetate as main biologically active constituents are known for their anti-inflammatory properties (Heidari et al., 2016). Previous studies also revealed anti-microbial effects of COR-EO against *Staphylococcus aureus* (Vasiee et al., 2016) which also held true for Gram-negative bacteria including *Escherichia coli*, *Pseudomonas* species (Dash et al., 2011; Liu et al., 2020), and enteropathogens such as *Salmonella* species (Kubo et al., 2004; Prakash et al., 2019) and *C. jejuni* (Salem et al., 2019). Furthermore, limonene and linalool were shown to increase the permeability of the bacterial cell membrane and to inhibit the cellular respiration resulting in cell death (Han et al., 2020; Guo et al., 2021). Hence, both, LEM-EO and COR-EO constitute promising candidates for antibiotics-independent intervention strategies in the combat of human campylobacteriosis.

Our previous preclinical placebo-controlled intervention studies provided evidence for anti-pathogenic and immune-modulatory properties of several EOs derived from clove (Bereswill et al., 2021), garlic (Heimesaat et al., 2021b), cardamom (Heimesaat et al., 2021a), and cumin (Mousavi et al., 2021) in an acute murine campylobacteriosis model applying secondary abiotic (SAB) IL-10 deficient (IL-10^−/−^) mice. The secondary depletion of the commensal murine gut microbiota by antibiotic pretreatment and the lack of the anti-inflammatory *il-10* gene are pivotal prerequisites for successful colonization of *C. jejuni* alongside the murine gastrointestinal tract and for the pathogen-induced pro-inflammatory immune responses mounting in acute enterocolitis (Bereswill et al., 2011; Haag et al., 2012). Within 6 days, *C. jejuni* infected SAB IL-10^−/−^ mice exhibit characteristic features of acute human campylobacteriosis such as bloody diarrhea and wasting, colonic mucosal aggregates of innate and adaptive immune cells and enhanced secretion of pro-inflammatory mediators in intestinal, but also in extra-intestinal and systemic organs (Haag et al., 2012). In the actual study, we used the SAB IL-10^−/−^ mouse model to assess the anti-pathogenic and/or immune-modulatory effects of LEM-EO and COR-EO in acute campylobacteriosis upon prophylactic application.

## 2. Materials and methods

### 2.1. Ethical statement

All experimental interventions were approved by the commission for animal welfare (“Landesamt für Gesundheit und Soziales,” LaGeSo, Berlin; registration number G0104/19) and done in accordance with the European animal welfare guidelines (2010/63/EU).

### 2.2. Secondary abiotic IL-10−/− mice

IL-10^−/−^ C57BL/6j mice were reared in the Forschungsinstitute für Experimentelle Medizin, Charité – Universitätsmedizin Berlin, Germany, maintained within an experimental semi-barrier under standard specific pathogen-free conditions and provided *ad libitum* access to autoclaved water and rodent food pellets (ssniff R/M-H, V1534-300, Sniff, Soest, Germany). For intestinal microbiota eradication, 3-week-old female and male mice were subjected to an antibiotic cocktail *via* the drinking water as reported previously (Heimesaat et al., 2006; Bereswill et al., 2011; Heimesaat et al., 2022). Following quality control by culture and Real time-PCR to confirm the absence of intestinal bacteria as described earlier (Bereswill et al., 2011), the resulting SAB mice were handled under aseptic conditions.

### 2.3. Prophylactic application of compounds

Prophylactic application of LEM-EO (LEM Peel EO, California origin, purchased from Sigma-Aldrich, Munich, Germany) or COR-EO (COR seed EO, from Sigma-Aldrich, Munich, Germany) was initiated 7 days before *C. jejuni* infection. The EOs were dissolved in 2.5 mL 2% carboxymethylcellulose (Sigma-Aldrich, Munich, German), and added to 17.5 mL sterile phosphate buffered saline (PBS, Thermo Fisher Scientific, Waltham, MA, United States) plus 80 mL autoclaved tap water (*ad libitum*). Assuming a mean weight of 25 g and an approximal daily drinking volume of 5 mL, mice were subjected to a drinking solution with a final concentration of 500 mg/L (LEM-EO) or 800 mg/L (COR-EO) and received daily dose of 100 mg (LEM-EO) or 160 mg (COR-EO) per kg body weight as reported earlier (Burdock and Carabin, 2009; Falls et al., 2018). The placebo control mice received vehicle only.

### 2.4. *Campylobacter jejuni* infection and gastrointestinal pathogen loads

Live *C. jejuni* strain 81–176 bacteria were obtained from thawed frozen stocks and cultivated on solid media (karmali agar; Oxoid, Wesel, Germany). Age and sex matched SAB IL-10^−/−^ mice (3-month-old littermates) were infected with 10^9^ colony forming units (CFU) of the pathogen on days 0 and 1 by oral gavage. The numbers of viable *C. jejuni* bacteria were determined in fecal samples every day post-infection (p.i.), and upon necropsy in intraluminal gastrointestinal samples that had been homogenized in sterile PBS (Thermo Fisher Scientific, Waltham, MA, United States). Serial dilutions of fecal and gastrointestinal samples were plated onto karmali agar plates (Oxoid, Wesel, Germany) and incubated for at least 48 h at 37°C under microaerophilic conditions as described previously (Bereswill et al., 2011). The detection limit of the enteropathogens was 100 CFU per gram sample.

### 2.5. Clinical outcome and sampling

Upon initiation of respective prophylactic treatment and furthermore, before and every day after infection, we quantitatively assessed the clinical signs in mice with clinical scores (Supplementary Table S1) as described earlier (Heimesaat et al., 2014). After sacrifice by carbon dioxide inhalation on day 6 p.i., cardiac blood was drawn and *ex vivo* biopsies from kidneys, mesenteric lymph nodes (MLN) and colon as well as luminal samples from stomach, duodenum, ileum, and colon were removed.

### 2.6. Histopathological analyses

Colonic *ex vivo* biopsies were immediately fixed in 5% formalin and embedded in paraffin. Sections (5 μm) were stained with hematoxylin and eosin (H&E), examined by light microscopy (100 × magnification), and the histopathological changes were quantitatively graded according to histopathological scores (Supplementary Table S2; Erben et al., 2014). Furthermore, apoptotic epithelial cells, macrophages and monocytes, T lymphocytes, and regulatory T cells were counted in colonic paraffin sections (5 μm) stained with distinct primary antibodies (Supplementary Table S3) as described in more detail earlier (Heimesaat et al., 2018). An independent investigator counted numbers of specifically stained cells by light microscopy. The average number of positively stained cells in each sample was determined within at least six high power fields (HPF, 0.287 mm^2^, 400 × magnification).

### 2.7. Pro-inflammatory mediators

Colonic samples (longitudinally cut strips of approximately 1 cm^2^, washed in PBS; Thermo Fisher Scientific, Waltham, MA, United States) and *ex vivo* biopsies from MLN (3 nodes) and the kidney (one half after the longitudinal cut) were transferred to 24-flat-bottom well plates (Thermo Fisher Scientific, Waltham, MA, USA) containing 500 μL serum-free RPMI 1640 medium (Thermo Fisher Scientific, Waltham, MA, USA) supplemented with penicillin (100 μg/mL; Biochrom, Berlin, Germany) and streptomycin (100 μg/mL; Biochrom, Berlin, Germany). After an 18-h incubation period at 37°C, culture supernatants and serum samples were tested for IL-6, interferon-γ (IFN-γ), and monocyte chemoattractant protein-1 (MCP-1) by the Mouse Inflammation Cytometric Bead Assay (CBA; BD Biosciences, Heidelberg, Germany) using a BD FACSCanto II flow cytometer (BD Biosciences, Heidelberg, Germany).

### 2.8. Statistics

Data were pooled from four independent experiments, and medians and significance levels were calculated using GraphPad Prism (version 9; San Diego, CA, USA). Normalization of data was assessed by the Anderson-Darling test. Multiple comparisons were performed using the Kruskal–Wallis test with Dunn’s post-correction (for not normally distributed data) and the one-way ANOVA with Tukey post-correction (for normally distributed data). Probability (p) values ≤0.05 were considered significant.

## 3. Results

### 3.1. Gastrointestinal *Campylobacter jejuni* colonization following prophylactic oral lemon or coriander essential oil application to infected IL-10^−/−^ mice

**Figure 1:**
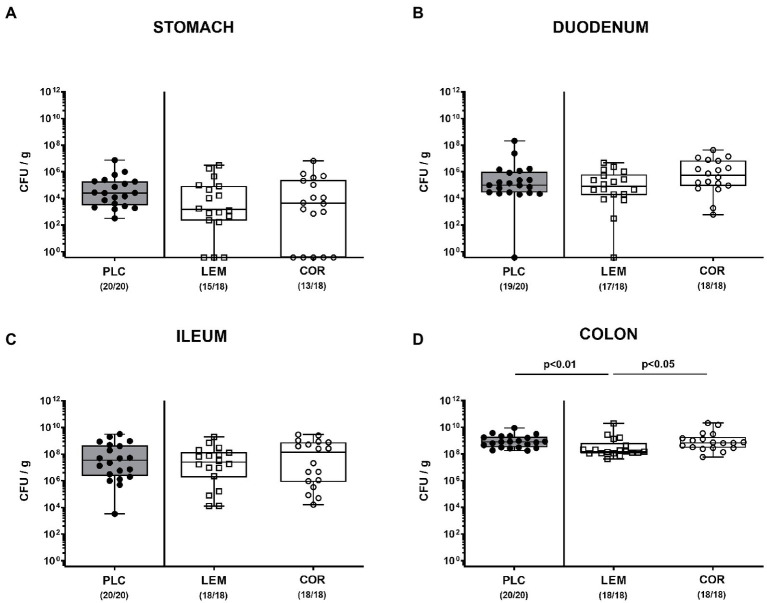
Gastrointestinal *C. jejuni* colonization following prophylactic oral application of lemon or coriander essential oil to infected IL-10^−/−^ mice. SAB IL-10^−/−^ mice were obtained as described in methods and subjected to prophylactic lemon (LEM; white squares) or coriander (COR; white circles) essential oil *via* the drinking water (*ad libitum*) starting 7 days prior infection. Placebo (PLC; black circles) control animals received tap water only. On days 0 and 1, mice were then perorally infected with *C. jejuni* strain 81–176 by gavage. The *C. jejuni* numbers (colony forming units per gram; CFU/g) were assessed in distinct gastrointestinal luminal samples from the **(A)** stomach, **(B)** duodenum, **(C)** ileum, and **(D)** colon taken following sacrifice of mice at day 6 post-infection. Box plots (indicating the 25th and 75th percentiles), whiskers (indicating the minimum and maximum values), medians (black bar inside box) and numbers of culture-positive mice out of the total number of analyzed animals (in parentheses) are shown. Significance levels (*p* values) were calculated by the Kruskal–Wallis test with Dunn’s post-correction using pooled data from four independent experiments.

SAB IL-10^−/−^ mice were perorally subjected to either oral LEM-EO or COR-EO *via* the drinking water starting from day 7 before infection. On days 0 and 1, mice were perorally infected with 10^9^ CFU of the *C. jejuni* strain 81–176. First, we addressed whether the prophylactic regimens had an impact on gastrointestinal colonization efficiencies of the pathogen. Our cultural analyses revealed comparable luminal *C. jejuni* loads in the stomach, the duodenum and the ileum upon necropsy (not significant (n.s.); Figures 1A–C), whereas approximately 0.5 log orders of magnitude lower median pathogen burdens were measured in the colonic lumen of LEM-EO as compared to COR-EO and placebo treated mice on day 6 p.i. (*p* < 0.05; Figure 1D). Hence, LEM-EO as opposed to COR-EO prophylaxis slightly lowered colonic *C. jejuni* loads.

### 3.2. Clinical outcome following prophylactic oral application of lemon or coriander essential oil to *Campylobacter jejuni* infected mice

**Figure 2:**
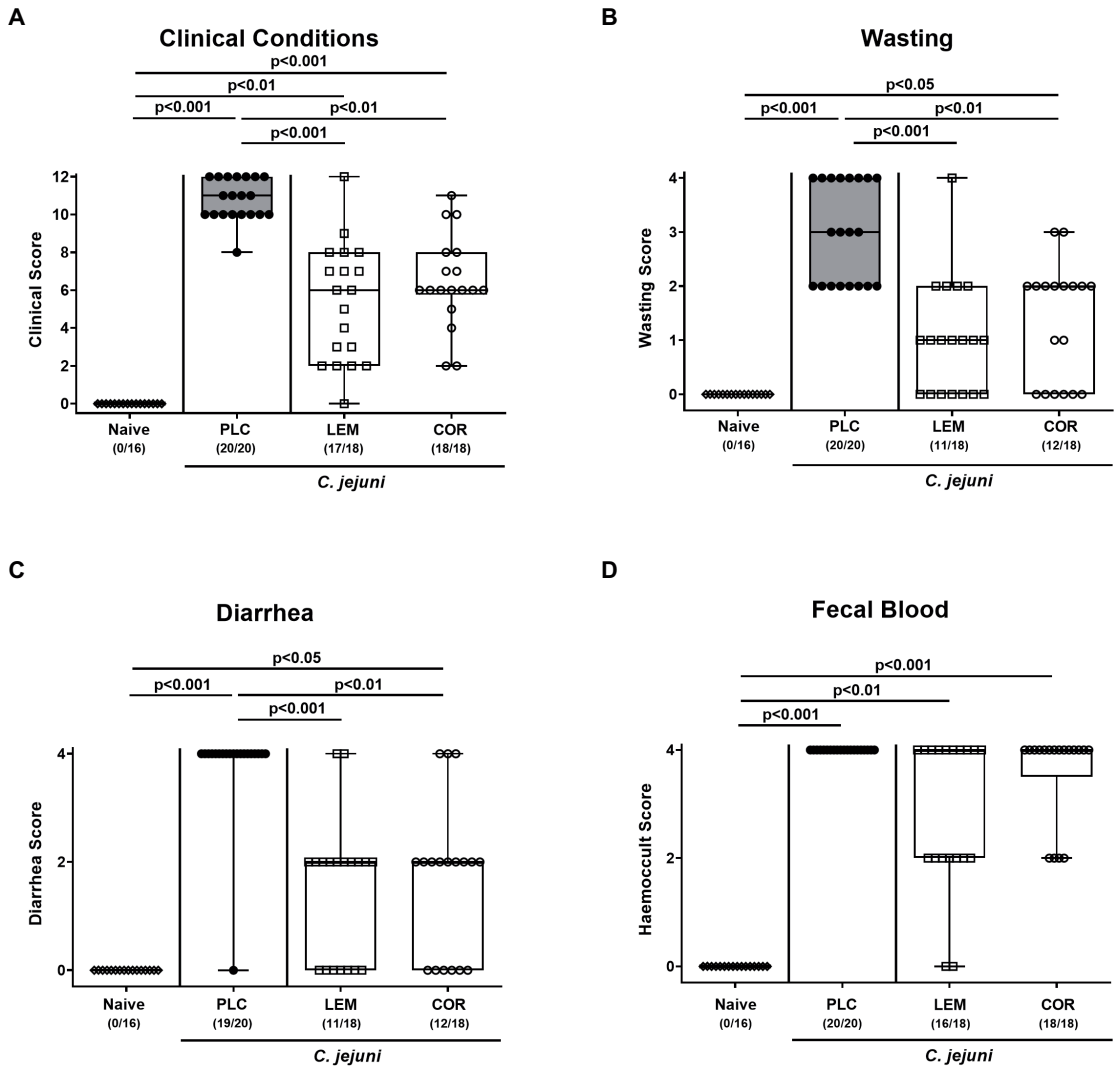
Clinical outcome following prophylactic oral application of lemon or coriander essential oil to *C. jejuni* infected mice. SAB IL-10^−/−^ mice were subjected to prophylactic lemon (LEM; white squares) or coriander (COR; white circles) essential oil *via* the drinking water (*ad libitum*) starting seven days prior infection. Placebo (PLC; black circles) control animals received tap water only. On days 0 and 1, mice were then perorally infected with *C. jejuni* strain 81–176 by gavage. **(A)** The overall clinical conditions of mice were quantitatively surveyed on day 6 post-infection by using a cumulative clinical scoring system (see methods) assessing **(B)** wasting symptoms, **(C)** diarrhea, and **(D)** fecal blood. Naive mice were included as non-infected, untreated controls (white diamonds). Box plots (indicating the 25th and 75th percentiles), whiskers (indicating the minimum and maximum values), medians (black bar inside box) and numbers of mice displaying respective clinical sign out of the total number of analyzed animals (in parentheses) are shown. Significance levels (*p* values) were calculated by the Kruskal–Wallis test with Dunn’s post-correction using pooled data from four independent experiments.

Next, we quantitatively surveyed the clinical outcome of infected mice upon either prophylactic regimen by using distinct clinical scores. On day 6 p.i., placebo treated mice exhibited acute campylobacteriosis characterized by wasting symptoms and bloody diarrhea and displayed median scores of 10 for the overall clinical outcome (Figure 2). Mice from both, the LEM-EO (*p* < 0.001) and COR-EO (*p* < 0.01) cohorts, however, were less distinctly compromised (Figure 2A) and exhibited lower scores for wasting and diarrhea in particular, when compared to placebo controls (p < 0.001 and p < 0.01; Figures 2B,C). Hence, both, LEM-EO and COR-EO prophylactic regimens alleviated clinical signs of acute murine campylobacteriosis.

### 3.3. Microscopic inflammatory sequelae in the colon following prophylactic oral application of lemon or coriander essential oil to *Campylobacter jejuni* infected mice

**Figure 3:**
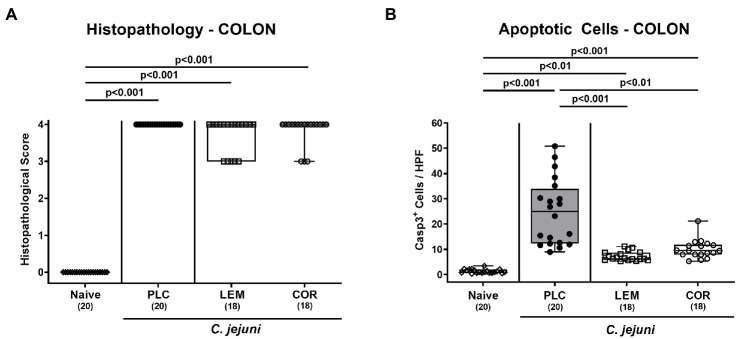
Microscopic inflammatory changes in the colon following prophylactic oral application of lemon or coriander essential oil to *C. jejuni* infected mice. SAB IL-10^−/−^ mice were subjected to prophylactic lemon (LEM; white squares) or coriander (COR; white circles) essential oil *via* the drinking water (*ad libitum*) starting 7 days prior infection. Placebo (PLC; black circles) control animals received tap water only. On days 0 and 1, mice were then perorally infected with *C. jejuni* strain 81–176 by gavage. **(A)** Colonic histopathological changes were surveyed on day 6 post-infection in hematoxylin and eosin-stained colonic paraffin sections by using histopathological scores. Furthermore, **(B)** numbers of apoptotic colonic epithelial cells were microscopically determined in six high power fields (HPF, 400 × magnification) of colonic paraffin sections positive for cleaved caspase-3 (Casp3^+^). Naive mice were included as non-infected, untreated controls (white diamonds). Box plots (indicating the 25th and 75th percentiles), whiskers (indicating the minimum and maximum values), medians (black bar inside box) and numbers of analyzed mice (in parentheses) are shown. Significance levels (*p* values) were calculated by the Kruskal–Wallis test with Dunn’s post-correction using pooled data from four independent experiments.

Further, we assessed the effects of both prophylactic applications on the microscopic inflammatory outcome of infected mice. Therefore, histopathological changes were determined in H&E stained colonic paraffin sections. When using established histopathological scores, mice from either treatment cohort displayed maximum median scores of 4 on day 6 p.i. indicative of severe histopathology (*p* < 0.001 versus naive; Figure 3A; Supplementary Figure S1A). Since apoptosis constitutes a reliable parameter for the grading of intestinal inflammatory morbidities including *C. jejuni* induced enteritis (Bereswill et al., 2022), we applied quantitative *in situ* immunohistochemistry to count colonic epithelial cells that were positive for cleaved caspase-3. In fact, *C. jejuni* infection resulted in marked increases in apoptotic colonic epithelial cells (*p* < 0.01–0.001 versus naive; Figure 3B; Supplementary Figure S1B). However, these increases were far less pronounced in mice prophylactically treated with either LEM-EO (*p* < 0.001) or COR-EO (*p* < 0.01) versus placebo (Figure 3B; Supplementary Figure S1B). Hence, both, prophylactic LEM-EO and COR-EO treatment alleviated pathogen-induced colonic epithelial cell apoptosis in *C. jejuni* infected mice.

### 3.4. Immune cell responses in the colon following prophylactic oral application of lemon or coriander essential oil to *Campylobacter jejuni* infected mice

**Figure 4:**
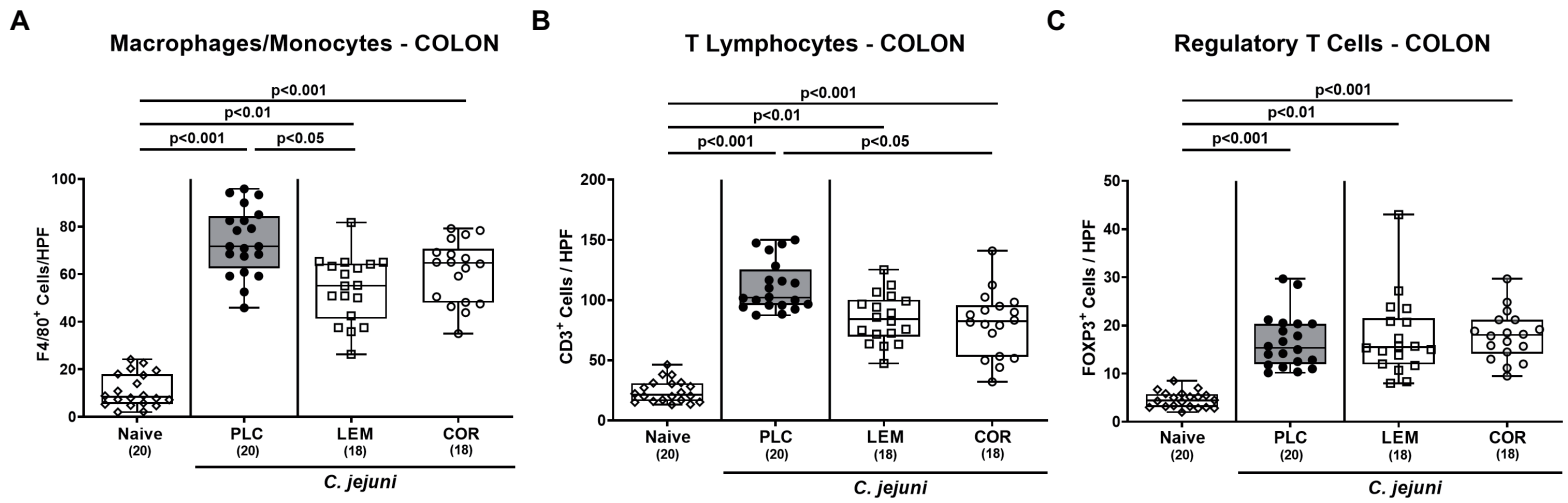
Immune cell responses in the colon following prophylactic oral application of lemon or coriander essential oil to *C. jejuni* infected mice. SAB IL-10^−/−^ mice were subjected to prophylactic lemon (LEM; white squares) or coriander (COR; white circles) essential oil *via* the drinking water (*ad libitum*) starting seven days prior infection. Placebo (PLC; black circles) control animals received tap water only. On days 0 and 1, mice were then perorally infected with *C. jejuni* strain 81–176 by gavage. On day 6 post-infection, the average numbers of **(A)** macrophages and monocytes (F4/80^+^), **(B)** T lymphocytes (CD3^+^), and **(C)** regulatory T cells (FOXP3^+^) per mouse were determined in immunohistochemically stained colonic paraffin sections from six high power fields (HPF, 400 × magnification). Naive mice were included as non-infected, untreated controls (white diamonds). Box plots (indicating the 25th and 75th percentiles), whiskers (indicating the minimum and maximum values), medians (black bar inside box) and numbers of analyzed mice (in parentheses) are shown. Significance levels (*p* values) were calculated by the one-way ANOVA test with Tukey post-correction **(A,B)** and the Kruskal–Wallis test with Dunn’s post-correction **(C)** using pooled data from four independent experiments.

Then, we tested whether prophylactic treatment with either EO impacted innate and adaptive immune responses upon *C. jejuni* infection. To address this, we quantitated defined innate and adaptive immune cell subsets in the colonic mucosa and lamina propria following immunohistochemical staining. On day 6 p.i., LEM-EO, but not COR-EO prophylactically treated mice displayed lower colonic numbers of F4/80 positive macrophages and monocytes as compared to placebo counterparts (*p* < 0.05; Figure 4A). Conversely, when staining with anti-CD3, T cell numbers were lower in the colon taken from COR-EO as opposed to LEM-EO challenged mice in comparison to the placebo group (*p* < 0.05; Figure 4B). A trend toward lower numbers of F4/80 positive and of CD3 positive cells was assessed in the colon of LEM-EO and COR-EO treated mice, respectively, when compared to placebo counterparts on day 6 p.i. (n.s. due to high standard deviations; Figures 4A,B). Furthermore, *C. jejuni* infection was associated with similar increases in Foxp3 positive regulatory T cell numbers in the colonic mucosa and lamina propria (*p* < 0.01–0.001 versus naive; Figure 4C). Hence, LEM-EO and COR-EO prophylaxis in *C. jejuni* infected mice resulted in differential innate and adaptive immune cell responses in the colon.

### 3.5. Intestinal pro-inflammatory cytokine secretion following prophylactic oral application of lemon or coriander essential oil to *Campylobacter jejuni* infected mice

**Figure 5:**
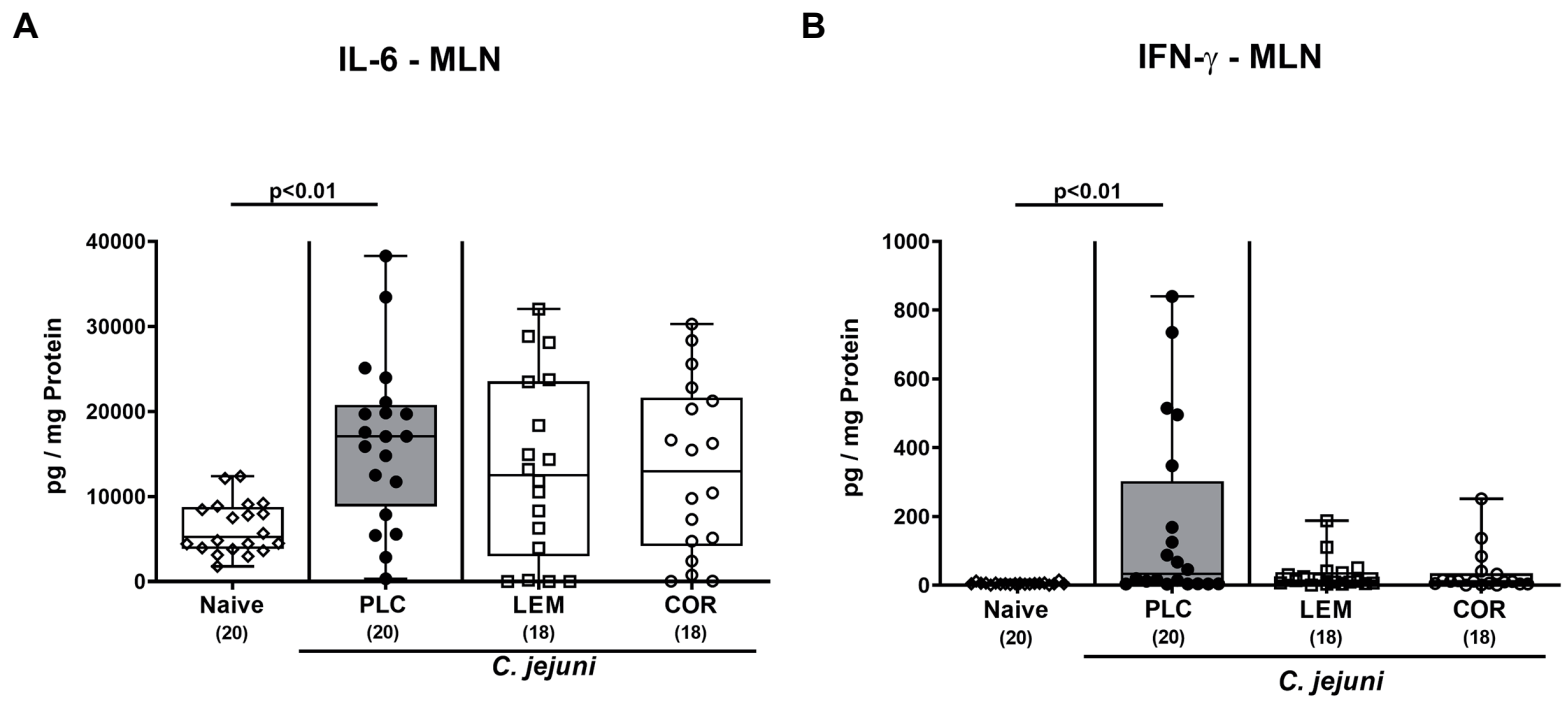
Intestinal pro-inflammatory cytokine secretion in mesenteric lymph nodes following prophylactic oral application of lemon or coriander essential oil to *C. jejuni* infected mice. SAB IL-10^−/−^ mice were subjected to prophylactic lemon (LEM; white squares) or coriander (COR; white circles) essential oil *via* the drinking water (*ad libitum*) starting seven days prior infection. Placebo (PLC; black circles) control animals received tap water only. On days 0 and 1, mice were then perorally infected with *C. jejuni* strain 81–176 by gavage. On day 6 post-infection, **(A)** IL-6 and **(B)** IFN-γ concentrations were measured in *ex vivo* biopsies derived from mesenteric lymph nodes (MLN). Naive mice were included as non-infected, untreated controls (white diamonds). Box plots (indicating the 25th and 75th percentiles), whiskers (indicating the minimum and maximum values), medians (black bar inside box) and numbers of analyzed mice (in parentheses) are shown. Significance levels (*p* values) were calculated by the Kruskal-Wallis test with Dunn’s post-correction using pooled data from four independent experiments.

Next, we measured the effects of LEM-EO and COR-EO prophylaxis on the pathogen-induced pro-inflammatory cytokine secretion in distinct intestinal compartments. *C. jejuni* infection was associated with increases in IL-6 and IFN-γ concentrations in the colon of mice from either treatment group (*p* < 0.01–0.001 versus naive; Supplementary Figure S2). In MLN, however, secretion of both, IL-6 and IFN-γ was enhanced following placebo application (p < 0.01; Figures 5A,B), whereas basal levels were measured in LEM-EO and COR-EO treated mice on day 6 p.i. (Figures 5A,B).

### 3.6. Extra-intestinal pro-inflammatory mediator secretion following prophylactic oral application of lemon or coriander essential oil to *Campylobacter jejuni* infected mice

**Figure 6:**
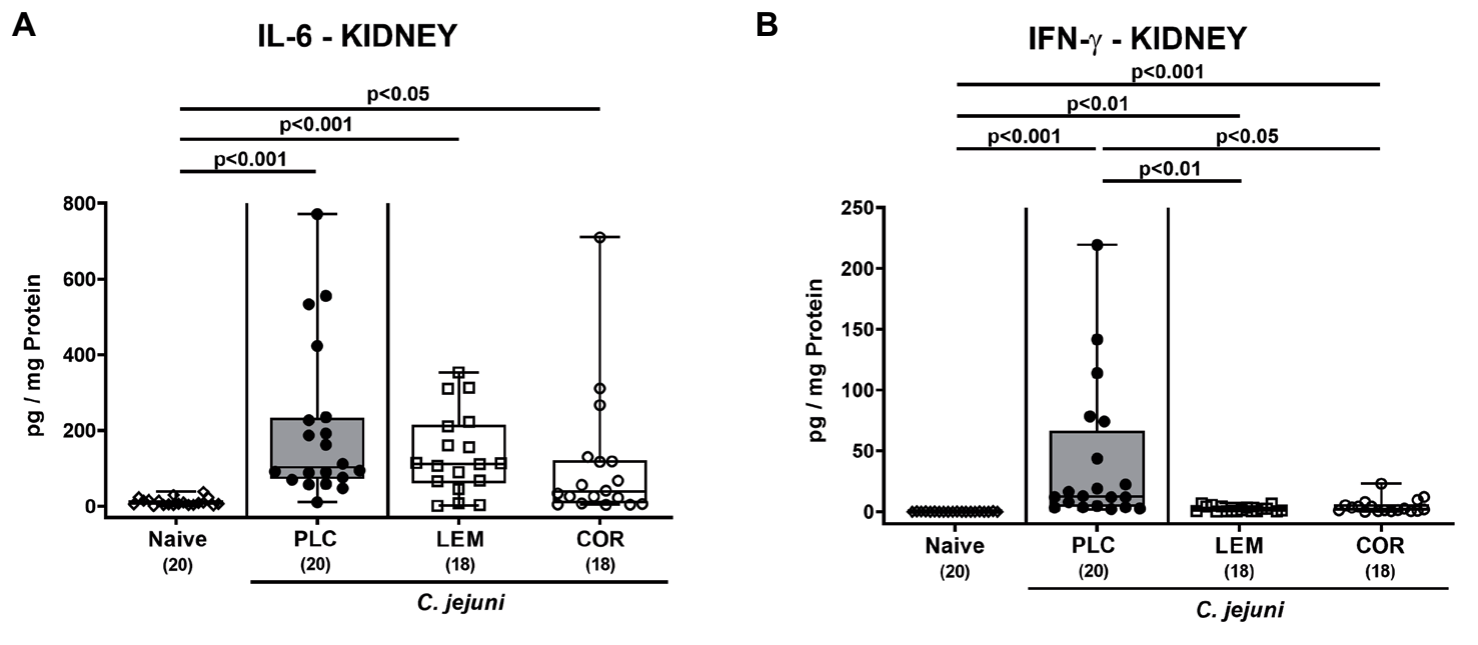
Pro-inflammatory cytokine secretion in the kidneys following prophylactic oral application of lemon or coriander essential oil to *C. jejuni* infected mice. SAB IL-10^−/−^ mice were subjected to prophylactic lemon (LEM; white squares) or coriander (COR; white circles) essential oil *via* the drinking water (*ad libitum*) starting seven days prior infection. Placebo (PLC; black circles) control animals received tap water only. On days 0 and 1, mice were then perorally infected with *C. jejuni* strain 81–176 by gavage. On day 6 post-infection, **(A)** IL-6 and **(B)** IFN-γ concentrations were measured in *ex vivo* biopsies derived from the kidneys. Naive mice were included as non-infected, untreated controls (white diamonds). Box plots (indicating the 25th and 75th percentiles), whiskers (indicating the minimum and maximum values), medians (black bar inside box) and numbers of analyzed mice (in parentheses) are shown. Significance levels (*p* values) were calculated by the Kruskal–Wallis test with Dunn’s post-correction using pooled data from four independent experiments.

Furthermore, we asked whether oral LEM-EO or COR-EO prophylaxis might exert anti-inflammatory effects in other organs of mice with acute campylobacteriosis. In fact, both IL-6 and IFN-γ secretion were enhanced in the kidneys from either group on day 6 p.i. (p < 0.05–0.001 versus naive; Figures 6A,B). In the case of IFN-γ, however, lower cytokine concentrations were measured following LEM-EO and COR-EO prophylaxis as compared to placebo application (p < 0.01 and p < 0.05, respectively; Figure 6B).

**Figure 7:**
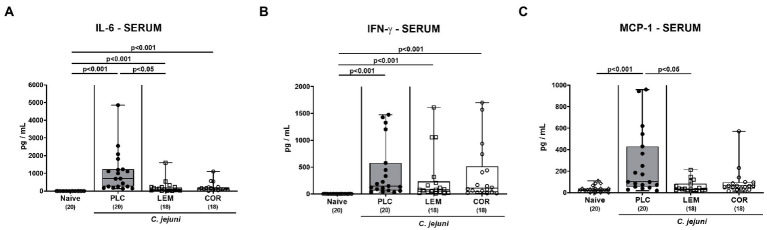
Systemic pro-inflammatory mediator secretion following prophylactic oral application of lemon or coriander essential oil to *C. jejuni* infected mice. SAB IL-10^−/−^ mice were subjected to prophylactic lemon (LEM; white squares) or coriander (COR; white circles) essential oil *via* the drinking water (*ad libitum*) starting 7 days prior infection. Placebo (PLC; black circles) control animals received tap water only. On days 0 and 1, mice were then perorally infected with *C. jejuni* strain 81–176 by gavage. On day 6 post-infection, **(A)** IL-6, **(B)** IFN-γ and **(C)** MCP-1 concentrations were measured in serum samples. Naive mice were included as non-infected, untreated controls (white diamonds). Box plots (indicating the 25th and 75th percentiles), whiskers (indicating the minimum and maximum values), medians (black bar inside box) and numbers of analyzed mice (in parentheses) are shown. Significance levels (*p* values) were calculated by the Kruskal-Wallis test with Dunn’s post-correction using pooled data from four independent experiments.

Next, we investigated systemic pro-inflammatory mediator secretion in serum samples taken on day 6 p.i. Enteropathogenic infection resulted in enhanced serum IL-6 and IFN-γ secretion in all groups (*p* < 0.001 versus naive; Figures 7A,B). Mice from the LEM-EO cohort, however, exhibited lower systemic IL-6 concentrations as compared to placebo controls (*p* < 0.05; Figure 7A), whereas at least a trend toward less distinct IL-6 secretion was assessed in serum samples from the COR-EO group versus the placebo cohort (n.s.; Figure 7A). Remarkably only the placebo control mice displayed increased MCP-1 concentrations in the serum samples taken on day 6 p.i. (*p* < 0.001 versus naive; *p* < 0.05 versus LEM-EO; Figure 7C), whereas basal levels were measured in the mice treated with either LEM-EO or COR-EO (n.s. versus naive; Figure 7C). Hence, prophylactic oral application of both, LEM-EO and COR-EO resulted in diminished secretion of distinct pro-inflammatory mediators in the kidneys and serum of *C. jejuni* infected mice.

## 4. Discussion

The worldwide increasing prevalence of *Campylobacter* infections has imposed serious challenges on healthcare and food safety (Neill, 2016; Robinson et al., 2016). The One Health concept aims at improving health-beneficial measures in humans and animals and addresses antibiotic resistance in foodborne diseases by interdisciplinary strategies (Robinson et al., 2016; McEwen and Collignon, 2018). In consequence, developing antibiotic-independent intervention strategies for the prophylaxis and treatment of human campylobacteriosis as well as finding alternative measures to limit antibiotic resistance not only in humans but also in livestock are utmost appreciable.

In our actual preclinical placebo-controlled intervention study we surveyed potential disease-alleviating effects of prophylactic oral LEM-EO and COR-EO application in an acute murine campylobacteriosis model. Neither treatment regimen resulted in relevant changes of the gastrointestinal pathogen loads. Even though on day 6 p.i., 0.5 orders of magnitude lower median *C. jejuni* numbers were determined in the colonic lumen of LEM-EO treated mice as compared to placebo counterparts, this difference might be considered as rather subtle and not biologically relevant given median colonic pathogen loads of more than 10^9^ viable *C. jejuni* bacteria per gram of colonic content. These results may have been unexpected since previous *in vitro* studies revealed anti-*Campylobacter* directed effects of both, LEM-EO and COR-EO (Duarte et al., 2016; Salem et al., 2019). Moreover, in our study, we applied the LEM-EO at a concentration of 500 mg/L that was far below the minimal inhibitory concentration (MIC) of 2,048 mg/L, whereas the COR-EO concentration of the drinking solution (800 mg/L) was higher than the measured MIC (512 mg/L). It is, however, highly likely that the intraluminal intestinal and hence, biologically active concentrations of the EOs were much lower due to mixing and diluting with the secretory intestinal fluids resulting in rather subtle anti-bacterial effects. One needs to further take into consideration that the composition of each EO includes a mixture of distinct terpenoids, terpens, and other aromatic and aliphatic components (Bakkali et al., 2008), that can be variable depending on the origin of plants, used tissue (peel, seeds, leaves, etc.) and different processing methods such as isolation and purification (Bassolé and Juliani, 2012). These factors have an important impact on the composition and thus, effectiveness of the EOs which makes it challenging to compare respective studies with each other. Therefore, we compared our results with those obtained from investigations using the major constituents of LEM-EO such as limonene and of COR-EO including linalool and linalyl acetate.

In our preclinical trial we show for the first time, that the prophylactic application of either EO improved the clinical outcome including wasting and diarrheal symptoms in mice with acute campylobacteriosis. In support, LEM peel powder treatment ameliorated dextran sulfate sodium (DSS) induced colitis in mice presenting with less severe weight loss and bloody diarrhea, if compared to placebo (Tinh et al., 2021). Additionally, in mice suffering from oxazolone-induced colitis, limonene administration changed the stool consistency from loose indicative for mild diarrhea to almost normal consistency resembling the effect of the reference drug sulfasalazine (Estrella et al., 2021). Interestingly, linalyl acetate inhibited olmesartan-induced intestinal hypermotility in rats (Kwon et al., 2018), and it is tempting to speculate that this might be a potential mechanism for the anti-diarrheal effect of exogenous COR-EO in *C. jejuni* infected IL-10^−/−^ mice.

The alleviated clinical signs in LEM-EO pretreated mice were paralleled by less distinct pathogen-induced colonic epithelial cell apoptosis. Although several investigations revealed pro-apoptotic effects of LEM in distinct cancer cells, two studies showed that the activation of caspase-3, which leads to apoptosis, is inhibited by LEM in the premature ovarian failure rat model (Mobasher et al., 2023) and in human astrocytes CCF-STTG1 (Koo et al., 2002), indicating that the pro- and anti-apoptotic effects of LEM are cell-type dependent. Furthermore, linalool derived from COR-EO has been shown to exert anti-apoptotic effects by the inhibition of caspase-3 and -9 attenuating ischemic injury in rodent PC12 cells (Hosseini et al., 2022). In our study, however, the pathogen-induced apoptotic responses in colonic epithelial cells were comparable in COR-EO pretreated and PLC control mice.

Furthermore, LEM-EO and COR-EO pretreatment prevented SAB IL-10^−/−^ mice from *C. jejuni*-induced increases in intestinal pro-inflammatory cytokine secretion as indicated by basal IL-6 and IFN-γ concentrations measured in MLN taken from the verum cohorts on day 6 p.i. In support, linalool pretreatment of mice that had been challenged with *Salmonella* Typhimurium endotoxin significantly lowered the IFN-γ concentrations in MLN (Lee et al., 2018).

Remarkably, the anti-inflammatory properties of both treatment regimens were not restricted to the intestinal compartment given that prophylactic LEM-EO and COR-EO application dampened IFN-γ concentrations in the kidneys of *C. jejuni* infected mice to basal levels. In support, exogenous LEM-EO protected rats and mice from nephrotoxicity that had been induced by aspirin and cisplatin, respectively (Bouzenna et al., 2016; Abdel-Daim et al., 2020), whereas COR derivatives were shown to enhance anti-oxidative capacities in the kidneys (Deepa and Anuradha, 2011) and to ameliorate arsenic-induced renal toxicity (Kumar et al., 2022). Strikingly, even systemic anti-inflammatory effects of the EO pretreatment regimens are underpinned by less distinct pro-inflammatory mediator concentrations measured in the serum samples taken from *C. jejuni* infected mice.

In conclusion, our preclinical placebo-controlled intervention study provides first evidence that LEM-EO and COR-EO constitute promising prophylactic measures to prevent the development of severe campylobacteriosis and the risk for post-infectious sequelae.

## Data availability statement

The original contributions presented in the study are included in the article/Supplementary material, further inquiries can be directed to the corresponding author.

## Ethics statement

The animal study was reviewed and approved by Landesamt für Gesundheit und Soziales (LaGeSo), Berlin.

## Author contributions

SM performed the experiments, analyzed the data, critically discussed results, and co-wrote the paper. DW performed the experiments and analyzed the data. SB provided advice in experimental design, critically discussed results, and co-wrote the paper. MH designed and performed the experiments, analyzed the data, critically discussed results, and wrote the paper. All authors contributed to the article and approved the submitted version.

## Funding

This work was supported by grants from the German Federal Ministries of Education and Research (BMBF) in frame of the zoonoses research consortium PAC-Campylobacter to MH and SB (IP7/01KI1725D and 01KI2007D) and from the Federal Ministry for Economic Affairs and Energy following a resolution of the German National Parliament, Deutscher Bundestag to MH and SB (ZIM, ZF4117908 AJ8). The funders had no role in study design, data collection and analysis, decision to publish or preparation of the manuscript.

## Conflict of interest

The authors declare that the research was conducted in the absence of any commercial or financial relationships that could be construed as a potential conflict of interest.

## Publisher’s note

All claims expressed in this article are solely those of the authors and do not necessarily represent those of their affiliated organizations, or those of the publisher, the editors and the reviewers. Any product that may be evaluated in this article, or claim that may be made by its manufacturer, is not guaranteed or endorsed by the publisher.
