# Supplementary material for: Disease alleviating effects following prophylactic lemon and coriander essential oil treatment in mice with acute campylobacteriosis
Source: Front Microbiol. 2023 Mar 29;14:1154407. doi: 10.3389/fmicb.2023.1154407 (PMC10090957; doi:10.3389/fmicb.2023.1154407)
Supplement: Supplementary file 1 [file Data_Sheet_1.pdf]

Supplementary Material

Disease alleviating effects following prophylactic lemon and coriander essential oil treatment in mice with acute campylobacteriosis

Soraya Mousavi, Dennis Weschka, Stefan Bereswill, Markus M. Heimesaat#

Correspondence: Markus M. Heimesaat; markus.heimesaat@charite.de

Supplementary Table S1: Clinical scoring (maximum 12 points)

Clinical aspect	Symptoms	Scores
**Wasting**	Normal	0
	Ruffled fur	1
	Less locomotion	2
	Isolation	3
	Severely compromised locomotion, pre-final aspect	4
**Stool consistency**	Formed feces	0
	Pasty feces	2
	Liquid feces	4
**Fecal blood**	No blood	0
	Microscopic detection of blood (Guaiac method using Haemoccult, Beckman, Coulter/PCD, Germany	2
	Macroscopic blood visible	4

Supplementary Table S2: Histopathological scoring (maximum 4 points)

Histopathology	Score
Minimal inflammatory cell infiltrates in the mucosa with intact epithelium	1
Mild inflammatory cell infiltrates in the mucosa and submucosa with mild hyperplasia and mild goblet cell loss	2
Moderate inflammatory cell infiltrates in the mucosa with moderate goblet cell loss	3
Marked inflammatory cell infiltration into the mucosa and submucosa with marked goblet cell loss, multiple crypt abscesses, and crypt loss	4

Supplementary Table S3: Primary antibodies for *in situ* immunohistochemical analyses

**Cells**	**Primary antibody**
Apoptotic epithelial cells	cleaved caspase-3 (Asp175, Cell Signaling, Beverly, MA, USA, 1:200)
Macrophages/monocytes	F4/80 (no. 14-4801, clone BM8, eBioscience, San Diego, CA, USA, 1:50
T lymphocytes	CD3 (no. N1580, Dako, 1:10)
Regulatory T cells	Foxp3 (clone FJK-165, no. 14-5773, eBioscience, 1:100)

Figure S1

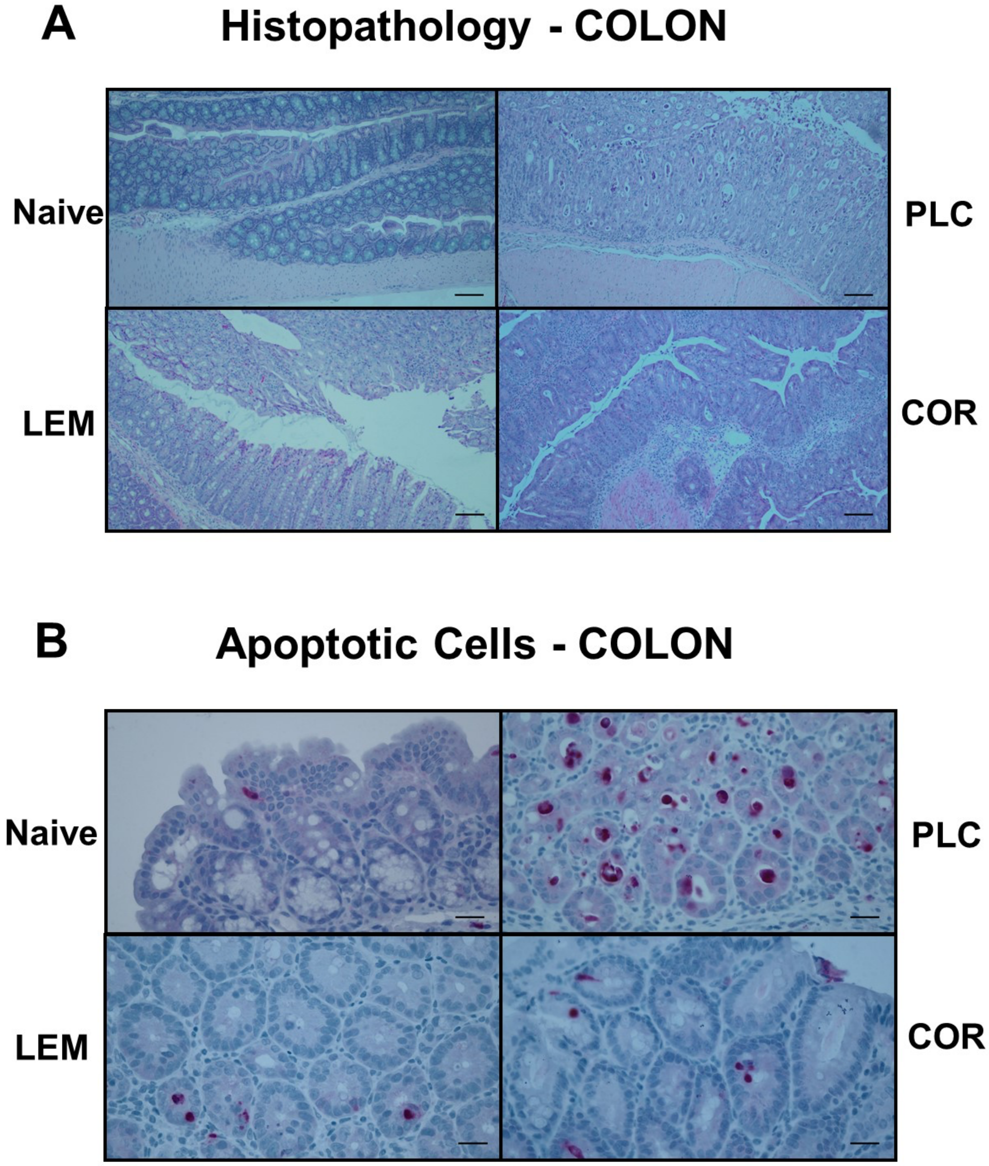

Supplementary Figure S1. Representative photomicrographs illustrating microscopic inflammatory changes in the colon following prophylactic oral application of lemon or coriander essential oil to *C. jejuni* infected mice. Secondary abiotic IL-$10^{-/-}$ mice were subjected to prophylactic lemon (LEM) or coriander (COR) essential oil via the drinking water (*ad libitum*) starting seven days prior to infection or received placebo (PLC). On days 0 and 1, mice were perorally infected with *C. jejuni* strain 81-176. Photomicrographs representative of four independent experiments illustrate **(A)** colonic histopathological changes on day 6 post-infection in hematoxylin and eosin-stained colonic paraffin sections (100x magnification; scale bar 100 µm) and **(B)** apoptotic colonic epithelial cells in large intestinal paraffin sections positive for cleaved caspase-3 (400x magnification, scale bar 25 µm). Naive mice were included as non-infected, untreated controls.

Figure S2

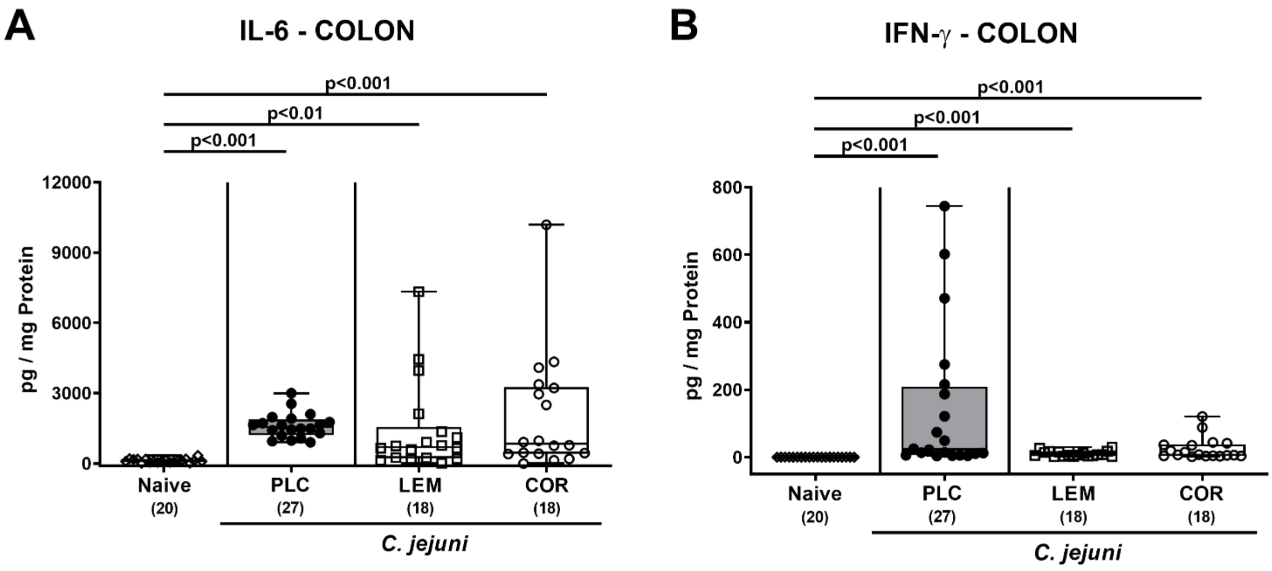

Supplementary Figure S2. Large intestinal pro-inflammatory cytokine secretion following prophylactic oral application of lemon or coriander essential oil to *C. jejuni* infected mice. Secondary abiotic IL-10$^{-/-}$ mice were subjected to prophylactic lemon (LEM; white squares) or coriander (COR; white circles) essential oil via the drinking water (*at libitum*) starting seven days prior to infection. Placebo (PLC; black circles) control animals received tap water only. On days 0 and 1, mice were then perorally infected with *C. jejuni* strain 81-176 by gavage. On day 6 post-infection, (**A**) IL-6 and (**B**) IFN-γ concentrations were measured in *ex vivo* biopsies derived from the colon. Naive mice were included as non-infected, untreated controls (white diamonds). Box plots (indicating the 25th and 75th percentiles), whiskers (indicating the minimum and maximum values), medians (black bar inside box) and numbers of analyzed mice (in parentheses) are shown. Significance levels (p values) were calculated by the Kruskal-Wallis test with Dunn's post-correction using pooled data from four independent experiments.
